# High throughput error correction in information reconciliation for semiconductor superlattice secure key distribution

**DOI:** 10.1038/s41598-021-82684-5

**Published:** 2021-02-16

**Authors:** Jianguo Xie, Han Wu, Chao Xia, Peng Ding, Helun Song, Liwei Xu, Xiaoming Chen

**Affiliations:** 1 Beijing Electronic Science and Technology Institute, Beijing, 100070 China; 2 Key Laboratory of Nanodevices and Applications, Suzhou Institute of Nano-Tech and Nano-Bionics, Chinese Academy of Sciences (CAS), Suzhou, 215123 China

**Keywords:** Computer science, Information technology

## Abstract

Semiconductor superlattice secure key distribution (SSL-SKD) has been experimentally demonstrated to be a novel scheme to generate and agree on the identical key in unconditional security just by public channel. The error correction in the information reconciliation procedure is introduced to eliminate the inevitable differences of analog systems in SSL-SKD. Nevertheless, the error correction has been proved to be the performance bottleneck of information reconciliation for high computational complexity. Hence, it determines the final secure key throughput of SSL-SKD. In this paper, different frequently-used error correction codes, including BCH codes, LDPC codes, and Polar codes, are optimized separately to raise the performance, making them usable in practice. Firstly, we perform multi-threading to support multi-codeword decoding for BCH codes and Polar codes and updated value calculation for LDPC codes. Additionally, we construct lookup tables to reduce redundant calculations, such as logarithmic table and antilogarithmic table for finite field computation. Our experimental results reveal that our proposed optimization methods can significantly promote the efficiency of SSL-SKD, and three error correction codes can reach the throughput of Mbps and provide a minimum secure key rate of 99%.

## Introduction

**Figure 1:**
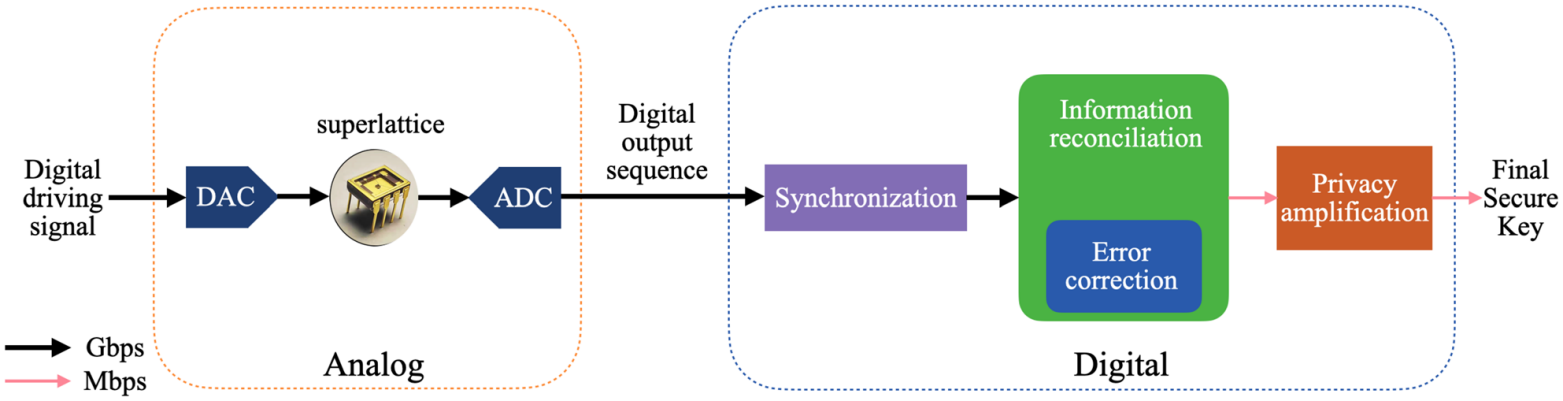
The procedure for generating final secure key using SSL-SKD.

Semiconductor superlattice secure key distribution (SSL-SKD) is a new secure key distribution technique based on chaos synchronization in superlattice PUF pairs^1^ driven by a synchronizing digital signal. SSL-SKD only uses the public channel with all electronic structures to create and provide secure key data for cryptography in unconditional security^2^. The procedure for generating the final secure key using SSL-SKD is divided into two phases, as shown in Fig. 1. In the analog front-end phase, the digital driving signal through a Digital-Analog Converter (DAC) was input to the superlattice device^3^, and the analog output of the superlattice was fed to an Analog-Digital Converter (ADC) to get a digital output sequence^4^. The analog front-end phase is now capable of operating at Gbps throughput^5^. Both of the sender and the recipient own one of the matched superlattice devices. Although the matched superlattices’ behavior can be very similar, they will generate slightly different digital signals due to inevitable differences of analog systems^2^.

**Figure 2:**
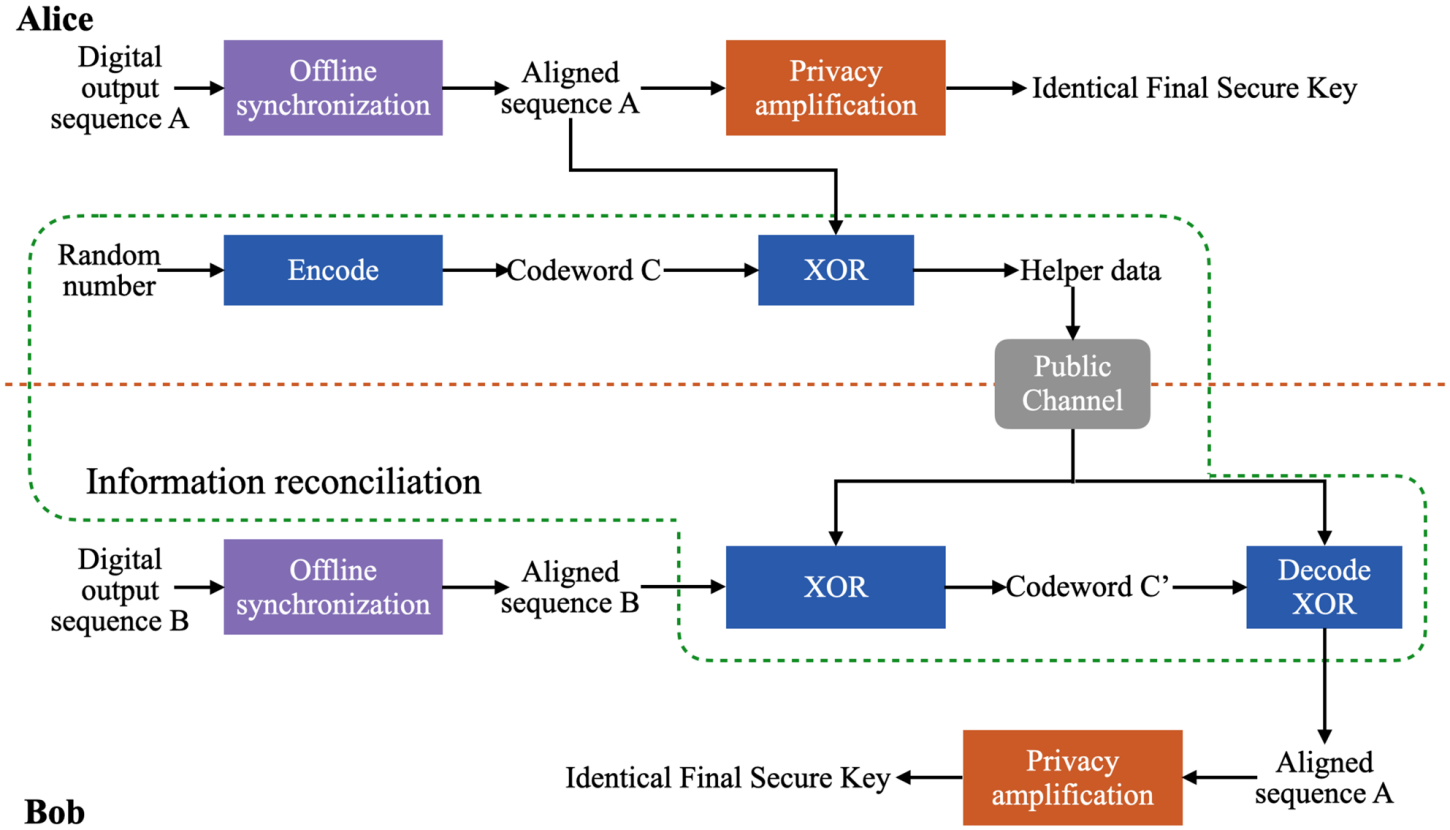
The post-processing procedure for generating identical final secure key using matched superlattice PUF pairs.

Thus, a digital backend phase called post-processing is needed. The main task of post-processing is to convert imperfect digital signals to consistent secure key pairs^6^. To accomplish this task, a series of post-processing operations have to be performed, including synchronization, information reconciliation, and privacy amplification, as shown in Fig. 2. Synchronization is to accurately map the digital output sequence of both parties. The information reconciliation technique is applied between the sender and the recipient^7^ to get identical output digital signals from both of the two superlattices. The goal of privacy amplification is to eliminate some of the key information that the attacker may obtain in post-processing, and generate a final secure key.

The confidentiality of SSL-SKD is ensured by the fact that semiconductor superlattices are good examples of strong physical unclonable functions (PUFs)^8^. Moreover, the secure keys are generated and used locally. Thus the secure keys are difficult to be reproduced by anyone else except the owner of the superlattice devices. Meanwhile, the high rate of the analog front-end will pave the way to the practical implementations of a one-time pad cipher. Based on the above researches, the principle and framework of the SSL-SKD system have been verified. The long-haul symmetric key distribution experiment based on superlattice pairs was successfully performed in the actual environment and got a preferable result^1^. The research for the SSL-SKD system has come to a practical period. We usually use error correction to perform information reconciliation in practical implementation, which is relatively computationally complex and viewed as the performance bottleneck.

Error correction is widely used in physical key distribution currently that allows for user authentication and encryption, e.g. Quantum Key Distribution (QKD)^9–11^ and Physical Unclonable Function (PUF)^12^. QKD is a technology based on the laws of quantum physics to create cryptographic keys between legitimate users^13^. It as a promising direction, solving the problem of key distribution has already taken a worthy place among systems that provide confidential information transmission^14^. BCH codes are typically used in small PUF systems^15,16^ due to their simple construction, easy implementation in resource-constrained situations. Recently, LDPC codes^17^ and Polar codes^18^ have entered our field of view due to their several advantages that can be summarized as follows: demonstrating better block error performance, error floors in much lower Bit Error Rate (BER) values, the ability to obtain good error performance with the length of block increases.

The error correction codes are closely related to the channel mathematical model by reliable channel transmission theorem^19^. We consider the slight differences in digital signals on matched superlattice PUF pairs is caused by binary symmetric channel (BSC) or AWGN channel using BPSK-mapper. Through the experiment, we found that the Hamming distance of matched superlattice PUF pairs is distributed between 3 and $12\%$, and the error distribution often appears in blocks. This high channel error rate makes it inconsistent with most error correction codes in the industry or academia. Moreover, it will bring high computational complexity and lower efficiency. It is necessary for a complete high throughput SSL-SKD system that the error correction must be able to operate at least Mbps to avoid limiting the throughput of final secure key distribution. The motivation of this paper is to design a method of error correction that makes the SSL-SKD system efficiency.

In this paper, we propose three high throughput error correction schemes by multi-threading and look-up table method in information reconciliation for SSL-SKD. We show that BCH codes, LDPC codes, and Polar codes with a $99\%$ secure key rate can perform the error correction step that determines the maximum key throughput for SSL-SKD. For BCH codes, we employ OpenMP^20^ for multi-codeword decoding, construct look-up tables to simplify the calculation of finite field. Eventually, the bit throughput achieves 2.7 Mbps. For LDPC codes, Distributed Stream Processing for the BP decoding by MPI^21^ was used to accelerate and reach 15 Mbps. For Polar codes, significant optimizations, including fixed-point arithmetic, a lookup-table implementation, and multi-codeword decoding, cause the bit throughput to reach 50 Mbps. It is confirmed that the SSL-SKD can be used for implementing one-time pad cipher. These three error correction codes are of great significance to SSL-SKD and play an important role in specific application scenarios.

## Results

### Distribution discrepancy of matched superlattice PUF pairs

Information reconciliation is an efficient way for matched superlattice PUF pairs to distill common corrected keys from similar digital output sequences. In Fig. 2, Alice (the encoder) calculates the syndrome information from its digital sequence and provides the helper data to Bob (the decoder). Then Bob uses helper data to correct its digital sequence so that the two parties have an identical digital sequence. In other words, the difference between aligned sequence *A* and *B* shifts to codeword *C* and $C'$. The process from codeword *C* to $C'$ can be view as the binary symmetric channel(BSC) or AWGN channel using BPSK-mapper at the transmitter and a hard-decision demapper at the receiver. The information reconciliation process is carried out as follows in detail.

On Alice’s side: $H=A+C$. And then transport helper data *H* to Bob through a public channel.

On Bob’s side: $C'=H+B=A+C+B=C+(A+B)=C+Noise$. After decoding, Bob obtains *C*, and extracts *A* from the operation $A=H+C$.

**Figure 3:**
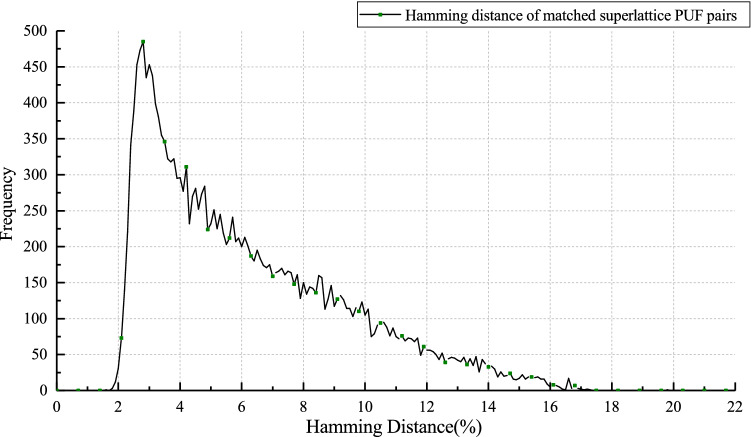
The Hamming distance of 20,000 blocks from matched superlattice PUF pairs. And the block-length is 10,000.

We quantize the original waveforms to obtain a binary sequence for our experiment. We did multiple experiments at room temperature. From those, we randomly select the number of blocks adding up to 20,000. As seen in Fig. 3, Hamming distance is mostly distributed between 3 and $12\%$. It is an important factor to determine the error correction code rate and also directly affects the final secure key rate. From the relationship between Bit Error Rate (BER) and Signal Noise Ratio (SNR) under BPSK modulation^22^, we can get the SNR value distributed between 2.2 and 4.0 after removing the BER value over $12\%$. The proportion of Hamming distance exceeding $12\%$ in the entire block does not exceed $5\%$ under multiple experiments. The frame error rate (FER) indicates the error correction performance, it refers to the failure probability of error correction. In order to ensure that the final secure key rate is not less than $99\%$, we set the FER target to $5e{-}3$. Since they will just be thrown away after the error correction, it is not crucial to lose these blocks for SSL-SKD.

Another vital characteristic of matched Superlattice PUF pairs is the burst-error^1^. The error distribution often appears in blocks rather than uniform. Therefore, when designing the error correction codes, we should try to increase the code length to reduce burst-error impact. The characteristics of high error rate and burst-error make the error correction of SSL-SKD different from other PUF systems. The implementation of the error correction schemes are evaluated on the multi-core computer; the specifications are shown in Table 1.

**Table 1:** Specifications of computer.

Parameter	Value
Operation system	Windows 10
CPU	Intel(R) I7-8700k
Threads per core	2
Memory	16GB LPDDR4
Storage	512GB SSD
Compiler	Microsoft Visual C++ 14.2
MPI	open mpi 1.10.7
OpenMP	Follow compiler

### Error correction with BCH codes

First we select BCH codes for SSL-SKD error correction. Since BCH code have excellent performance when the code length is short. Its structure is simple and easy to implement in resource-constrained situations. Most of the current PUF systems use BCH codes for error correction. There is a strict algebraic structure among the code length *n*, the length of information digits *k*, and the number of error correction digits *t*. For any positive integers $m\ge 3$ and $t<2^{m-1}$, there exists a binary BCH code with the parameters shown in Eq. (1). To minimize the impact of burst error, we select the code length of $n=4095$. This is a compromise between computational complexity and code length. According to Eq. (1), it is known that to satisfy $12\%$ error correction capability, the length of information digits we can only choose is $k=334$ or less. While $k=322$, the error correction capability can achieve $16.7\%$, which is $4\%$ higher than when $k=334$. $4\%$ of the difference can significantly improve the final secure key rate. Moreover, the proportion of Hamming distance exceeding $16.7\%$ does not exceed $0.1\%$. Thus, we finally choose the BCH code $(n,k,t)=(4095,322,682)$. The Berlekamp–Massey algorithm is selected to perform the decoding with low computational complexity and the benefit of software implementation.

1 $$\begin{aligned} \left\{ \begin{array}{l} n=2^m-1 \\ n-k \le mt \\ d_{min}\ge 2t+1 \end{array} \right. \end{aligned}$$

**Table 2:** By evaluating the 120,000 blocks with $(n,k,t)=$ (4095,322,682), the bit throughput of using MCD and the bit throughput of not using MCD are respectively obtained. We also show the CPU efficiency of using MCD and the frame errors.

Number of codewords	1	5	0	20	100	500	120,000
Bit throughput of using MCD (Mbps)	0.049	0.28	0.44	0.73	2.184	2.82	2.86
Bit throughput of not using MCD (Mbps)	0.58	0.61	0.63	0.64	0.63	0.64	0.65
CPU usage efficiency	8.93%	12.77%	20.45%	73.21%	85.21%	88.19%	90.91%
Frame errors	0	0	0	0	0	1	113
FER	–	–	–	–	–	–	9.42*e*−4

In the encoder, we calculate the generator polynomial in advance and design a generator polynomial table to avoid a mass of redundant computation. The look-up table method takes up more RAM space to reduce time consumption. In the decoder, we use OpenMP for parallel processing of codewords decoding to maximize CPU usage efficiency. Multi-codeword decoding (MCD) uses multiple CPU threads to decode multiple codewords simultaneously. Since the complicated calculation is based on the finite field, we construct the logarithmic table and antilogarithmic table with $m=12$ in Shared memory. As shown in Table 2, using MCD is significantly faster than not using MCD with the number of codewords increases. After the number of codewords reaches 500, the bit throughput of using MCD achieved about 2.82 Mbps. In contrast, the maximum bit throughput without using MCD is 0.64 Mbps (The CPU efficiency achieved more than $80\%$ when using OpenMP). Its decoding result $FER=9.42e{-4}$ match the target FER. In other words, while the error correction capability is $16.7\%$ with using BCH codes, the secure key rate exceeds $99\%$.

### Error correction with LDPC codes

A higher speed error correction method is required to support SSL-SKD. We select Low-Density Parity Check (LDPC) codes for SSL-SKD error correction due to several factors. Firstly, LDPC codes are a class of linear block codes with implementable decoders, which provide near-capacity performance on a broad set of channels^23^. Then there is a huge advantage of LDPC codes that possess sparse parity-check matrices. The LDPC code-construction techniques can be partitioned into random construction and mathematical construction. Compared to mathematical construction, randomly constructed LDPC codes have higher error correction capabilities and fewer iterations because they have larger loops on Tanner graphs. Avoiding trapping sets during decoding would often make the decoding converge faster and lower their error floors.

We generate the parity check matrices for $N/M=7200/6000$, $N/M=9000/8000$, $N/M=9000/7200$ and $N/M=16{,}200/13{,}500$ respectively. This construction method is designed by MacKay^24^. Besides, the smallest loops of the Tanner graph corresponding to these matrices is greater than 6. We will see that this sparseness characteristic makes the code amenable to various iterative decoding methods based on Tanner graphs, which provide near-optimal performance in many instances. For parallel computing, we choose belief propagation (BP) decoding algorithm, which iteratively updates message between variable nodes (VNs) and check nodes (CNs) to converge on valid codewords.

**Table 3:** Four kinds of LDPC Codes are evaluated with 120,000 blocks.

Codewords	$N=7200,M=6000$	$N=9000,M=8000$	$N=9000,M=7200$	$N=16{,}200,M=13{,}500$
Bit throughput of using MPI (Mbps)	16.17	15.77	15.34	15.19
Bit throughput of not using MPI (Mbps)	4.08	4.14	4.16	4.78
CPU usage efficiency	88.77%	85.53%	89.16%	90.11%
Number of iterations	5	4	5	3
Frame errors	1045	139	1261	803
FER	8.7*e*−3	1.16*e*−3	1.05*e*−2	6.69*e*−3

The number of iterations is the average value after 120,000 blocks of codeword measurements. The CPU usage efficiency is the peak value that appears when codewords were evaluated. Take the bit throughput of average value by multiple measurements.

To maximize the bit throughput, we construct lookup tables for the generator matrix ${\mathbf{G}}$ and parity check matrix ${\mathbf{H}}$, respectively. These two matrices are stored in Shared memory while the program is running. The calculation of the BP algorithm with time complexity $O(N\log N)$ at CNs to update value is complicated. Thus we practice the improved BP algorithm (Min-Sum algorithm^25^) to calculate log-likelihood ratio (LLR) values. Distributed Stream Processing uses MPI to perform BP decoding. In Stream Processing, a single vector value from a stream of multiple vectors is computed in a distributed manner. The data within each vector is distributed among multiple processors that perform computations, and then the value is gathered in the master processor. In the encoder, matrix multiplications are distributed across the processors and eventually converge to the master processor to perform addition and output. In the decoder, we perform the LLR values update for VNs and CNs on multiple processors.

For these four kinds of LDPC codes, 120,000 codewords are tested. The bit throughput of using MPI, the bit throughput of not using MPI, the CPU usage efficiency, the number of uncorrectable codewords, the number of iterations were shown in Table 3. The bit throughput with MPI exceeds 15 Mbps and is approximately four times faster than without MPI. However, as the block size increases, the memory required to store data is significantly increased, and the bit throughput is slightly attenuated. Under the same code rate, error correction capability is enhanced as the code length increases, and the number of iterations is also decreasing. This situation shows the excellent performance of LDPC codes with long code-length. The decoding results for LDPC code with $N/M=9000/8000$ can match the target FER and provide the secure key rate by more than $99\%$. Additionally, the bit throughput is about 5.5 times of BCH code above-mentioned. Experiments show that the optimized implementations of LDPC codes can provide 15 Mbps secure key throughput for SSL-SKD.

### Error correction with polar codes

The use of Polar codes has been considered for QKD^26^ previously. Many specificities make Polar codes suitable for SSL-SKD post-processing. Firstly, Polar codes are the first provably capacity achieving family of codes with low encoding and decoding complexity. Secondly, they are as easy to construct as BCH codes. Furthermore, an impressive feature of Polar codes is their regular recursive structure. It allows us to implement a recursive, successive-cancellation (SC) decoder that achieves a higher speed than LDPC codes by software. For a given noise level on a given channel, Density Evolution^27^ allows us to compute the capacities of the different bits of the code. Some of the bits corresponding to channels with the lowest capacities are revealed and are called the frozen bits (usually 0) of the codeword. Based on the SNR value calculated above, we use the Density Evolution to calculate the position of the frozen bit and set $R=\frac{K}{N}=\frac{1}{4}$.

**Table 4:** Six kinds of Polar codes are evaluated with 120,000 blocks.

Codewords ($R=\frac{1}{4}$)	$N=4096$	$N=8192$	$N=16{,}384$	$N=32{,}768$	$N=65{,}536$	$N=131{,}072$
Bit throughput of using MCD (Mbps)	72.63	64.33	61.77	56.61	53.27	50.64
Bit throughput of not using MCD (Mbps)	12.77	12.14	11.60	10.85	9.93	9.36
CPU usage efficiency	89.87%	85.72%	83.42%	84.77%	89.71%	93.20%
Frame errors	1432	1351	663	294	147	117
FER	1.19*e*−2	1.13*e*−2	5.53*e*−3	2.45*e*−3	1.23*e*−3	9.75*e*−4

The number of threads MPI opens is 8. The CPU usage efficiency is the peak value that appears when codewords are evaluated. Take the bit throughput of average value by multiple measurements.

An important optimization in this decoder is to use fixed-point arithmetic and a lookup-table implementation of the function $\varphi (x)=\log (\tanh (x/2))$ used to update log-likelihood ratios (LLRs). Furthermore, we use MPI technology to perform multi-codeword decoding (MCD) for Polar codes. When the number of threads MPI opens is 8, it can guarantee the fastest speed and the highest CPU usage efficiency. Each thread independently obtains some codewords that need to be corrected to perform error correction procedures. The lookup table is in Shared memory, and the master thread allocates the data to be processed by the sub-thread. Polar code is inherently suitable for software implementation, making it much faster than the BCH codes and LDPC codes.

Under the condition of $R=\frac{1}{4}$, we evaluate six different block sizes $N = 4096$, $N = 8192$, $N = 16{,}384$, $N = 32{,}768$, $N = 65{,}536$, $N = 131{,}072$. For these six kinds of Polar codes, 120,000 codewords are tested. The bit throughput of using MCD, the bit throughput of without MCD, the CPU usage efficiency, the number of uncorrectable codewords are shown in Table 4. After MPI opens eight threads, its CPU usage efficiency reaches the highest, and bit throughput is also maximum. From the view of frame errors, when the code rate of Polar codes is greater than that of LDPC codes, the error correction ability is stronger than that of LDPC codes. As the code length increases, the long-length performance of the Polar code gradually becomes prominent. On the other hand, the bit throughput is gradually decreasing. The decoding results for Polar code with $N\ge 32{,}768$ can match the target FER and provide the secure key rate by more than $99\%$. Additionally, the bit throughput is about 17.7 times of BCH code above-mentioned. Experiments show that the optimized implementations of Polar codes can provide exceeding 50 Mbps secure key throughput and secure key rate $99\%$ minimum for SSL-SKD. The Polar codes decoding throughput by the software is enough for state-of-the-art SSL-SKD implementations.

**Table 5:** Throughput comparison with QKD by different type of error correction codes.

Refs.	Code type	Block length	Code rate	Platforms	Throughput (Mbps)
Jouguet et al.^26^	MET-LDPC	$2^{17}$	0.1	GPU	7.3
Milicevic et al.^28^	QC-LDPC	$2^{20}$	0.1	GPU	9.17
Jouguet et al.^26^	Polar	$2^{16}$	0.1	CPU	10.9
This work	BCH	4095	0.079	CPU	2.86
This work	MacKay-LDPC	9000	$\frac{1}{9}$	CPU	15.77
This work	Polar	$2^{17}$	0.25	CPU	50.64

## Discussion

In this paper, we propose an experiment of multi-threaded high bit throughput error correction for SSL-SKD. Different from many communication channels, QKD or traditional PUF, the Hamming distance between matched superlattice PUF pairs ranges from 3 to 12% (relatively high), and the error pattern is burst-error. We show that BCH codes, LDPC codes, and Polar codes with a $99\%$ secure key rate minimum and exceeding the throughput of Mbps can be used to perform the error correction step for SSL-SKD. The performance of the proposed optimized scheme and the results obtained by other work are shown in Table 5. BCH code is used for feasibility verification of the post-processing scheme in QKD with the performance omitted^29^. In our implementation of SSL-SKD, the bit throughput of BCH code with parameters $(n,k,t)=(4095{,}322{,}682)$ reaches 2.8 Mbps, but the code rate ($R = 0.079$) is extremely low into an uncommon realm relative to other published works^30–32^. Jouguet et al. respectively obtain the speed to 7.3 Mbps with MET-LDPC code on GPU and 10.9 Mbps with Polar code on CPU^26^. Milicevic et al. obtain the speed to 9.17 Mbps with quasi-cyclic (QC) LDPC codes^28^ on GPU. For LDPC codes, we perform fine-grained parallelization for the Min-Sum algorithm. Good performance has been obtained of LDPC codes on multi-core CPU with bit throughput using MPI reaches 15 Mbps. For Polar codes, we select multi-codeword decoding, in which the main thread controls the IO and communicates with sub-threads to reduce the delay caused by IO to the greatest possible extent. Then, Polar codes achieve good efficiencies with bit throughput of using MCD reaches 50 Mbps. The error correction speed we achieved is faster than previous demonstrations, which is supporting high throughput SSL-SKD system.

The optimized scheme we proposed can be applied in different scenarios with great efficiency. One of the key features of the BCH code is that there is precise control over the number of symbol errors correctable by the code during code design. It simplifies the design of the decoder for these codes, using small, low-power electronic hardware. BCH codes are of great significance for the miniaturization of SSL-SKD. Both LDPC and Polar codes can meet the practical application requirements of SSL-SKD, and they both have the advantage of high speed and close to the Shannon limit. Moreover, we have seen that the Polar decoders that can match the error-correction performance of LDPC codes usually have lower hardware efficiency than their LDPC decoder counterparts. The low hardware efficiency stems mainly from the low throughput that SC decoder is not suitable for parallel computing, and not so much from their area requirements. This is the first practical application of Polar code for physical key distribution as far as we know. Polar codes are typically used in the demonstration of SSL-SKD. LDPC codes are now very mature in communication; its hardware implementation and chip design have entered the industry. Soon as SSL-SKD, the hardware implementation of LDPC codes in information reconciliation will match the analog stage throughput, and finally, the throughput of SSL-SKD can reach Gbps.

## Methods

### BCH encoder and decoder

Algebraic coding is the feature of BCH codes. To encode a binary sequence of length *k* bit, first write it as a polynomial $m(x)=m_{0}+m_{1}x+m_{2}x^{2}+\cdots +m_{k-1}x^{k-1}$. Through generator polynomial *g*(*x*) we can get the polynomial of the check bit $r(x)=x^rm(x)modg(x)$. Through *r*(*x*) and *m*(*x*) then the codeword polynomial $c(x)=x^rm(x)+x^rm(x)modg(x)$. At this point, the encoding is completed, and the error codeword information *R*(*x*) is received on the decoding party.

Then, syndrome $S=\{s_{1},s_{2},\ldots ,s_{2t}\}$ is calculated. The error-locator polynomial $\sigma (x)=\sigma _{t}x^{t}+\sigma _{t-1}x^{t-1}+\cdots +\sigma _{1}x+1$ can be found by Berlekamp–Massey^33^ algorithm under the syndrome *S*. Last solving the roots of the polynomial $\sigma (x)$ by Chien search^34^ to determine the error location. The decoding algorithm as shown in Algorithm 1. 
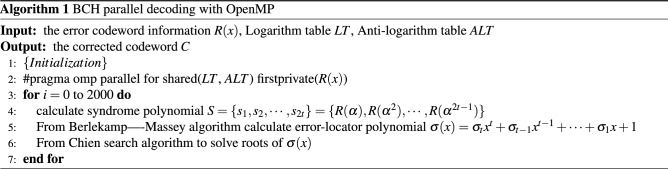


### LDPC encoder and decoder

For encoding, the information bits are first copied to the output bits. Each processor selects a subset of columns to form the *k* columns of Generator matrix $\mathbf{G}$. The partial output calculated by each processor is then gathered at the master processor by using **MPI_Gatherv()** command from MPI for further processing.

**Figure 4:**
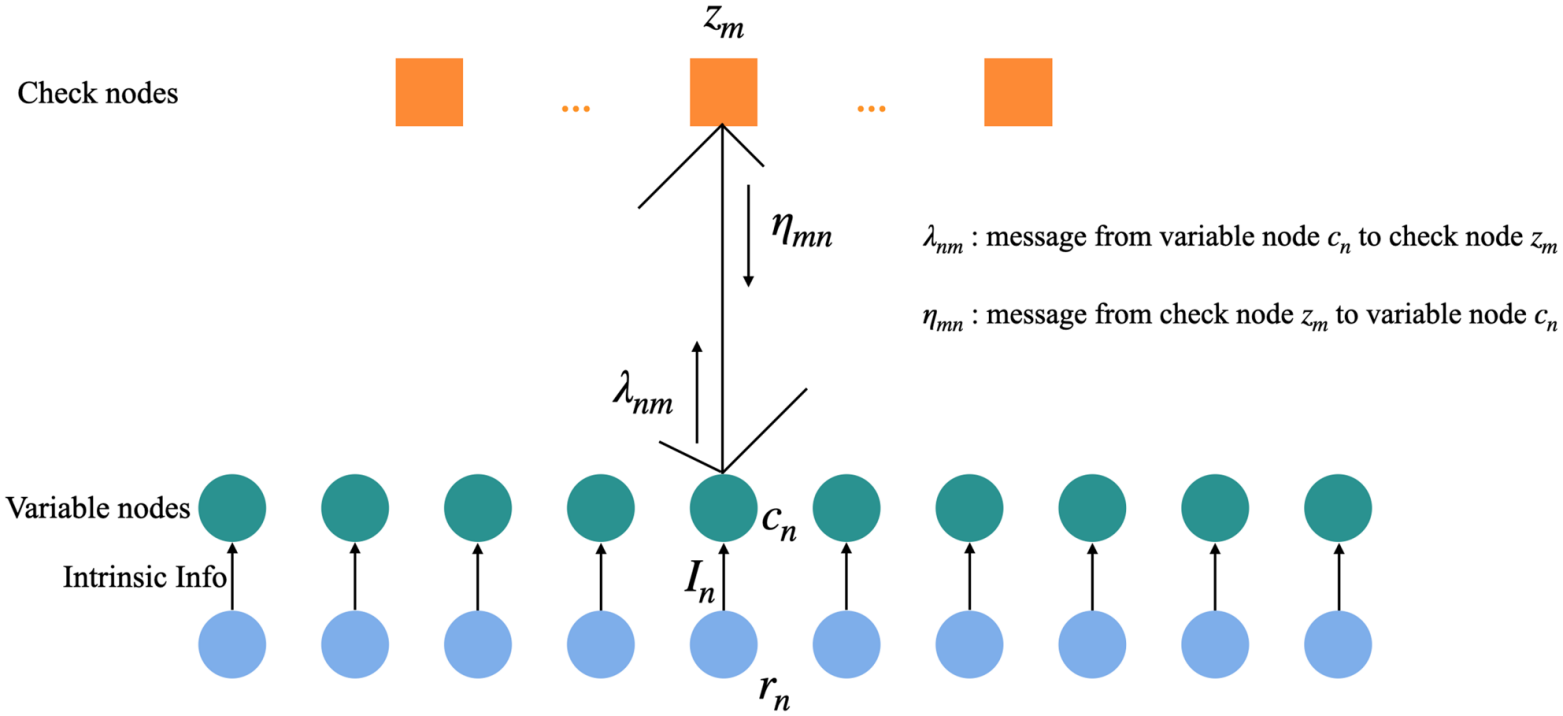
Variable node and check node transfer information mutual. Intrinsic Information is defined $I_{n}=\log \frac{p(r_{n}|c_{n}=0)}{p(r_{n}|c_{n}=1)}$. $I_{n}$ is the characteristic of channel. For BSC channel, if $r_{n}=0$, $I_{n}=\log \frac{1-p}{p}$, if $r_{n}=1$, $I_{n}=\log \frac{p}{1-p}$. For AWGN channel, $I_{n}=\log \frac{-2\sqrt{E_{b}}}{\sigma ^{2}}x$, $\sqrt{E_{b}}$ is the modulated signal amplitude.

For Min-Sum decoding algorithm, the information passed between the variable nodes (VNs) and the check nodes (CNs) are the log-likelihood ratio information (LLR) as shown in Fig. 4. The LDPC decoding process using the Min-Sum decoding algorithm is divided into four parts. First initialize $\eta _{mn}^{(0)}=\lambda _{nm}^{(0)}=0$, $\lambda _{nm}^{(1)}=I_{n}$. Then the VNs are updated at the *k*-th iteration using Eq. (2).

2 $$\begin{aligned} \lambda _{nm}^{(k)}=I_{n}+\sum _{m'\in M_{n,m}}\eta _{m'n}^{(k-1)} \end{aligned}$$

The next CNs are updated at the *k*-th iteration using Eq. (3).

3 $$\begin{aligned} \eta _{mn}^{(k)}=\prod _{n'\in N_{m,n}}sgn\left( \eta _{mn'}^{(k)}\right) \min _{n'\in N_{m,n}}\left| \lambda _{mn'}^{(k)}\right| \end{aligned}$$

Afterwards, determine whether $\lambda _{n}^{(k)}=I_{n}+\sum _{m\in M_{n}}\eta _{mn}^{(k)}$ is greater than 0, if $\lambda _{n}^{(k)} \ge 0$ is satisfied, $c_{n}=0$, if $\lambda _{n}^{(k)} < 0$ is satisfied, $c_{n}=1$. Finally, judge whether to stop the iteration. If $cH^{T}=0$ is satisfied, the decoding stops, otherwise the iteration is continued.

For each iteration of the decoding algorithm, we first divide the VNs and CNs among all CPU processors. For each iteration the processors calculate the LLR $\eta _{mn}^{(k)}$ of CNs using Eq. (3). When all processors have calculated their respective updated values, the CNs send the updated value to adjacent VNs. In the same way, the VNs’ values are updated and sent. The steps of node update are separated for all CPU processors that ensures the minimum communication consumption.

### Polar encoder and decoder

Polar code is a new linear block code based on the channel polarization theory. Channel polarization refers to the combination and splitting of any $N=2^n(n\ge 0)$ independent BDMCs in a specific way. As the number of channels N increases, the sub-channel characteristics show a polarization phenomenon. According to the phenomenon of channel polarization, *N* original channels that are mutually independent can be transformed into *N* channels with unequal channel capacity. When *N* tends to infinity, some channels’ capacity tends to 0, and others tend to 1. Assuming that the capacity of *K* channels tends to 1, and the $N-K$ channels tends to 0. *K* channels with capacity close to 1 can be selected to transmit information bits, and $N-K$ channels with a capacity close to 0 are selected to transmit frozen bits. Thereby realizing a correspondence from *K* information bits to *N* codewords, that is, realizing the encoding process of the Polar code with $R=\frac{K}{N}$. The specific encoding method of Polar code can be expressed by $x_{1}^{N}=u_{1}^{N}G_{N}$, where $G_{N}=B_{N}F^{\otimes n}$, $B_{N}$ is the *N*-order bit permutation matrix, $$F=\begin{bmatrix} 1 &{}\quad 0 \\ 1 &{}\quad 1 \end{bmatrix}$$.

SC decoding algorithm is not suitable for parallel implementation, so we use MPI technology for multi-codeword decoding. The decoding party received error codeword $y_{1}^{N}$. The decoding process is to obtain an estimation of $\hat{u}_{1}^{N}$ of the information sequence $u_{1}^{N}$ based on the known received signal $y_{1}^{N}$. *A* denote the set of information bit positions, and the complement $A^{c}$ denote the set of frozen bit positions. The SC decoding is shown in Eq. (4).

4 $$\begin{aligned} \hat{u}_{1}^{N}={\left\{ \begin{array}{ll} h_{i}\left( y_{1}^{N},\hat{u}_{1}^{i-1}\right) &{}\quad i \in A\\ u_{i}&{}\quad i\in A^{c} \end{array}\right. } \end{aligned}$$

$$h_{i}(y_{1}^{N},\hat{u}_{1}^{i-1})={\left\{ \begin{array}{ll} 0 &{}\quad L_{N}^{(i)}(y_{1}^{N},\hat{u}_{1}^{i-1})\ge 1\\ 1 &{}\quad {\text{others}} \end{array}\right. }$$ is the decoding criterion, where $L_{N}^{(i)}=\frac{W_{N}^{(i)}(y_{1}^{N},\hat{u}_1^{i-1}|0)}{W_{N}^{(i)}(y_{1}^{N},\hat{u}_{1}^{i-1}|1)}$.

While the block size of Polar code tends to infinity, since each split channel is close to full polarization, the SC decoding algorithm can ensure the correct decoding of each information bit, so that the Polar code can theoretically reach the symmetric capacity of the channel *I*(*W*).
